# Supplementary material for: Integrated Oxygen Consumption Rate, Energy Metabolism, and Transcriptome Analysis Reveal the Heat Sensitivity of Wild Amur Grayling (Thymallus grubii) Under Acute Warming
Source: Biology (Basel). 2025 Dec 1;14(12):1718. doi: 10.3390/biology14121718 (PMC12730672; doi:10.3390/biology14121718)
Supplement: Supplementary file 1 [file biology-14-01718-s001.zip › biology-4007856-supplementary.pdf]

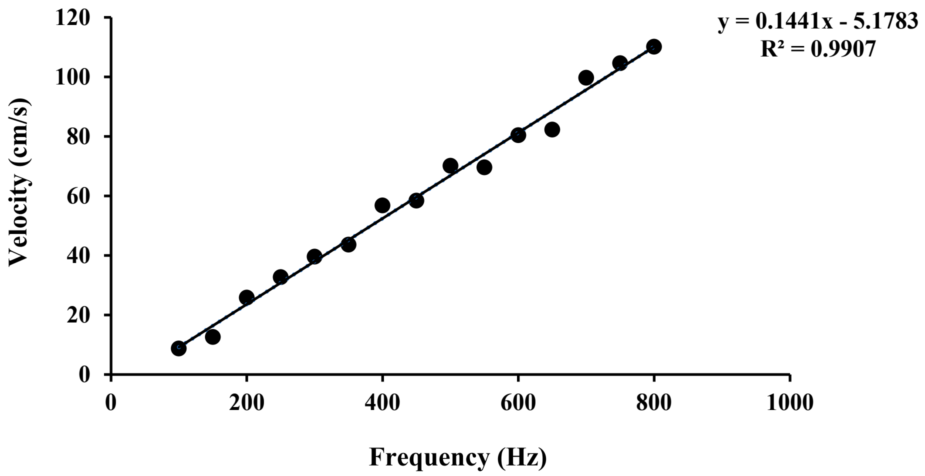


**Figure S1.** Relationship between flow velocity in the experimental tank measured by tachometer and frequency adjusted by the controller.

**Table S1.** The primer sequence of qRT-PCR.

| Gene | Primer sequence | Product Length (bp) |
|---|---|---|
| *slc39a8* | F:CGCCCTTCCTCATCTCTTGG | 171 |
| | R:GGTGGCATAGAACAGAGGGG | |
| *vtg3* | F: TAGCTCTTCCTGCCAGACCT | 203 |
| | R: AGCCCACTCCTGAACCATTG | |
| *sult2b1* | F:TGGGTTGTCCTGGTTGTGG | 180 |
| | R:GTGCGTTGCATCTTCCAGAAA | |
| *zp* | F:TAGATGGCAAAGGCGGATGT | 216 |
| | R:TGCTCCAGTTTACTCAGCGA | |
| *paps* | F:TAGGTTGTTCAGACGGCAGG | 154 |
| | R:AGTTGAATCAGGGTTCCGGG | |
| *bsp4* | F:GGCCATGCTTTGCGATCTG | 84 |
| | R:TGAGGAGGCGGTTCTGAAG | |
| *btn2a2* | F:GGAGAGAAGGGTGCGTTTCA | 200 |
| | R:TTCATGGTAGACGCTGCACA | |
| *kcnmb4* | F:ACCAGGAACTTCAGTGTCGG | 223 |
| | R:CCTGACTTCTCCTCCCTCCT | |
| *sh2d5* | F: TTCATCCTGCTAAAGGGACCG | 91 |
| | R: CTTACTCCACACCAGAGCCTT | |

**Table S2.** Quality control of the RNA-seq data obtained from different samples.

| Sample | Raw Reads | Raw Bases | Clean Reads | Clean Bases | Error Rate | Q20 | Q30 | GC Content |
|---|---|---|---|---|---|---|---|---|
| con1 | 44488880 | 6.67G | 41051764 | 6.16G | 0.03% | 97.06% | 92.40% | 45.30% |
| con2 | 44656232 | 6.69G | 40831172 | 6.12G | 0.03% | 97.02% | 92.32% | 46.19% |
| con3 | 50398792 | 7.55G | 45930550 | 6.89G | 0.03% | 97.16% | 92.62% | 47.01% |
| C15-1 | 43637782 | 6.54G | 39423198 | 5.91G | 0.03% | 96.92% | 92.15% | 44.51% |
| C15-2 | 42478682 | 6.37G | 38142912 | 5.72G | 0.03% | 97.13% | 92.51% | 44.81% |
| C15-3 | 41484764 | 6.22G | 37560160 | 5.63G | 0.03% | 97.12% | 92.53% | 44.26% |
| C21-1 | 46223832 | 6.9G | 45789492 | 6.8G | 0.01% | 98.87% | 96.49% | 46.09% |
| C21-2 | 44627758 | 6.7G | 44227470 | 6.6G | 0.01% | 98.92% | 96.63% | 46.46% |
| C21-3 | 44834200 | 6.7G | 44390106 | 6.6G | 0.01% | 98.85% | 96.46% | 46.02% |

**Table S3.** Statistical of database annotation.

| Database | Number of Unigenes | Percentage(%) |
|---|---|---|
| Annotated in NR | 72403 | 48.84 |
| Annotated in eggNOG | 53508 | 36.10 |
| Annotated in SwissProt | 46471 | 31.35 |
| Annotated in Pfam | 37509 | 25.30 |
| Annotated in GO | 51687 | 34.87 |
| Annotated in KEGG | 50335 | 33.96 |
| Annotated in all Databases | 73392 | 49.51 |
| Total Unigenes | 148233 | 100 |

**Table S4.** The detailed description of DEGs and KEGG pathway associated with energy metabolism.

| | Gene | Name | P-value | Change | KEGG pathway |
|---|---|---|---|---|---|
| C15vsCon | hk | Hexokinase | 0.15 | up | glycolysis/gluconeogenesis |
| | sdh | Succinate dehydrogenase | 0.92 | up | citrate cycle (TCA cycle) |
| | idh | Isocitrate dehydrogenase | | down | |

| | | | | | |
|---|---|---|---|---|---|
| | aco | Aconitate hydratase | | down | |
| | fasn | Fatty acid synthase, animal type | 0.39 | up | Fatty acid biosynthesis |
| | hadh | 3-hydroxyacyl-CoA dehydrogenase | 0.08 | down | Fatty acid degradation |
| | hadha | Enoyl-CoA hydratase/long-chain 3-hydroxyacyl-CoA dehydrogenase | | down | |
| C21vsCon | fasn | Fatty acid synthase, animal type | 0.43 | down | Fatty acid biosynthesis |
| | hadh | 3-hydroxyacyl-CoA dehydrogenase | 1.51 | up | Fatty acid degradation |
| | hadha | Enoyl-CoA hydratase/long-chain 3-hydroxyacyl-CoA dehydrogenase | | up | |

**Table S5.** Individual pathway IDs of KEGG pathway in **Figure 5**.

| **Figure 5c** | **Figure 5d** |
|---|---|
| map04141(Protein processing in endoplasmic reticulum) | map00190 (Oxidative phosphorylation) |
| map03060 (Protein export) | map04932 (Non-alcoholic fatty |

| | |
|---|---|
| | liver disease) |
| map00100 (Steroid biosynthesis) | map03040 (Spliceosome) |
| map00250 (Alanine, aspartate and glutamate metabolism) | map00260 (Glycine, serine and threonine metabolism) |
| map05204 (Chemical carcinogenesis - DNA adducts) | map05415 (Diabetic cardiomyopathy) |
| map05140 (Leishmaniasis) | map05208 (Chemical carcinogenesis - reactive oxygen species) |
| map00510 (N-Glycan biosynthesis) | map04146 (Peroxisome) |
| map05142 (Chagas disease) | map05022 (Pathways of neurodegeneration - multiple diseases) |
| map00983 (Drug metabolism - other enzymes) | map03010 (Ribosome) |
| map05134 (Legionellosis) | map05012 (Parkinson disease) |
| map04612 (Antigen processing and presentation) | map04714 (Thermogenesis) |
| map04260 (Cardiac muscle contraction) | map05020 (Prion disease) |
| map04080 (Neuroactive ligand-receptor interaction) | map00071 (Fatty acid degradation) |
| map04145 (Phagosome) | map04610 (Complement and coagulation cascades) |
| map00260 (Glycine, serine and threonine metabolism) | map00280 (Valine, leucine and isoleucine degradation) |
| map04978 (Mineral absorption) | map03018 (RNA degradation) |
| map04380 (Osteoclast differentiation) | / |
| map00900 (Terpenoid | / |

| | | |
|---|---|---|
| backbone biosynthesis) | | |
| / | / | |
| / | / | |

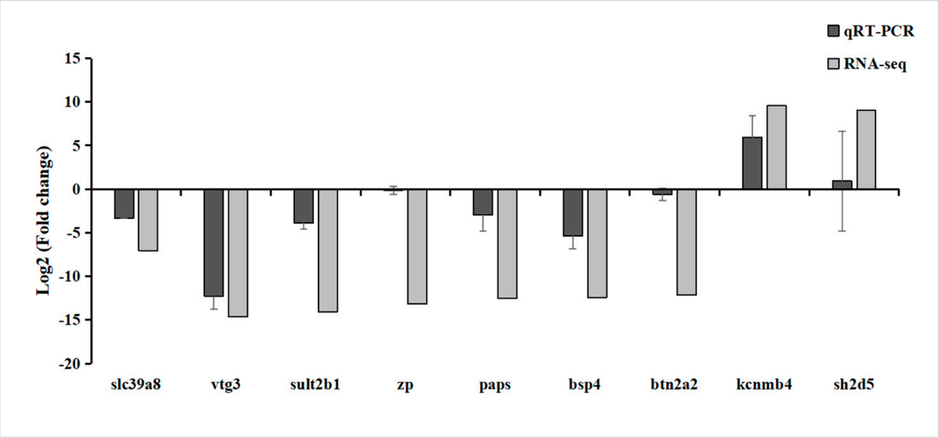


**Figure S2.** qRT-PCR validation for the RNA-seq results (n=3). X-axis indicated the gene name while Y-axis represented the $\log_2$ (Fold change) value of each DEGs. qRT-PCR results were calculated by $2^{-\Delta\Delta CT}$ method.
